# A Possible Role of Tetrodotoxin-Sensitive Na^+^ Channels for Oxidation-Induced Late Na^+^ Currents in Cardiomyocytes

**DOI:** 10.3390/ijms25126596

**Published:** 2024-06-15

**Authors:** Anja Schneider, Axel Hage, Inês Carvalheira Arnaut Pombeiro Stein, Nils Kriedemann, Robert Zweigerdt, Andreas Leffler

**Affiliations:** 1 Department of Anesthesiology and Intensive Care Medicine, Hannover Medical School, 30625 Hannover, Germanyhage.axel@mh-hannover.de (A.H.); stein.ines@mh-hannover.de (I.C.A.P.S.); 2 Leibniz Research Laboratories for Biotechnology and Artificial Organs (LEBAO), Department of Cardiothoracic, Transplantation and Vascular Surgery (HTTG), REBIRTH—Research Center for Translational Regenerative Medicine, Hannover Medical School, 30625 Hannover, Germany

**Keywords:** nitroxyl, ROS, oxidative stress, Nav1.5, Nav1.3, cardiomyocyte, late current

## Abstract

An accumulation of reactive oxygen species (ROS) in cardiomyocytes can induce pro-arrhythmogenic late Na^+^ currents by removing the inactivation of voltage-gated Na^+^ channels including the tetrodotoxin (TTX)-resistant cardiac α-subunit Nav1.5 as well as TTX-sensitive α-subunits like Nav1.2 and Nav1.3. Here, we explored oxidant-induced late Na^+^ currents in mouse cardiomyocytes and human-induced pluripotent stem cell-derived cardiomyocytes (hiPSC-CMs) as well as in HEK 293 cells expressing Nav1.2, Nav1.3, or Nav1.5. Na^+^ currents in mouse cardiomyocytes and hiPSC-CMs treated with the oxidant chloramine T (ChT) developed a moderate reduction in peak current amplitudes accompanied by large late Na^+^ currents. While ChT induced a strong reduction in peak current amplitudes but only small persistent currents on Nav1.5, both Nav1.2 and Nav1.3 produced increased peak current amplitudes and large persistent currents following oxidation. TTX (300 nM) blocked ChT-induced late Na^+^ currents significantly stronger as compared to peak Na^+^ currents in both mouse cardiomyocytes and hiPSC-CMs. Similar differences between Nav1.2, Nav1.3, and Nav1.5 regarding ROS sensitivity were also evident when oxidation was induced with UVA-light (380 nm) or the cysteine-selective oxidant nitroxyl (HNO). To conclude, our data on TTX-sensitive Na^+^ channels expressed in cardiomyocytes may be relevant for the generation of late Na^+^ currents following oxidative stress.

## 1. Introduction

Voltage-gated sodium channels are responsible for the rapid upstroke of the action potential in cardiomyocytes [1], and several α-subunits of voltage-gated Na^+^ channels are expressed in ventricular cardiomyocytes. While Nav1.5 is the predominant cardiac subunit being crucial for the proper generation of action potentials, the brain-type TTX-sensitive α-subunits Nav1.1, Nav1.3, and Nav1.6 were reported to account for approximately 25% of Na^+^ currents in mammalian cardiomyocytes and to regulate cardiac contractility and rhythmogenesis [2,3]. Nav1.5 is predominantly localized to the intercalated discs, the lateral membrane, and to a lesser extent in cardiac t-tubules [4]. Nav1.1, Nav1.3, and Nav1.6 are localized to t-tubules or near t-tubule openings [5,6]. Kirchhof et al. reported that a peptide that activates Nav1.3 and Nav1.6 can induce positive inotropy without provoking arrhythmias [7]. We have previously demonstrated that Nav1.5 and brain-type subunits are differentially inhibited Na^+^ channel inhibitors including local anesthetics [8]. Brain-type subunits may also be relevant for the emergence and treatment of arrhythmias [9,10,11], and they were suggested to be relevant for the emergence of pro-arrhythmic non-inactivating late Na^+^ currents in cardiomyocytes [12,13,14,15]. While late Na^+^ currents have been described for several chronic pathologies, they can also rapidly develop in the course of acute ischemia associated with an intracellular accumulation of reactive oxidative species (ROS) [16]. Early reports described that hydrogen peroxide induces late Na^+^ currents in ventricular cardiomyocytes [17]. These late currents at least partly originate from a removal of fast inactivation of Na^+^ currents, a ROS-induced effect that was demonstrated to be due to an oxidation of intracellular methionine residues conserved in all α-subunits [18]. When considering that Nav1.5 is the predominant cardiac α-subunit, however, it seems intriguing that most reports show that ROS induce a prominent reduction in Na^+^ currents generated by Nav1.5 [19,20,21,22]. This ROS-induced inhibition of Nav1.5 is due to an oxidation of the pore cysteine residue C373 [18,23]. This residue was also shown to dictate the intermediate TTX resistance of Nav1.5 [24,25], and Nav1.5 is the only subunit with a cysteine at this position [1]. Brain-type α-subunits with a phenylalanine or tyrosine at the corresponding site, are highly TTX-sensitive [25]. Furthermore, oxidants were demonstrated to induce slightly increased peak current amplitudes as well as large persistent currents on Nav1.4, Nav1.7, and Nav1.8 [18,26]. Thus, different α-subunits seem to display distinct ROS sensitivities, a yet rather poorly investigated property as compared to other well-described functional differences among α-subunits [1].

We hypothesized that if brain-type α-subunits are expressed in cardiomyocytes, they should account for a significant proportion of oxidant-induced late Na^+^ currents. To address this question, we performed whole-cell patch clamp recordings on neonatal mouse cardiomyocytes and hiPSC-derived cardiomyocytes as well as on recombinant Nav1.2, Nav1.3, and Nav1.5 channels expressed in HEK 293 cells. Late Na^+^ currents were induced by the strong oxidant chloramine T (ChT) as well as by UVA-light. We also investigated the effects induced by nitroxyl (HNO), a cysteine-selective oxidant known to induce positive inotropy without provoking arrhythmias [27].

## 2. Results

### 2.1. Late Na^+^ Currents Induced by the Strong Oxidant Chloramine T 

**Figure 1:**
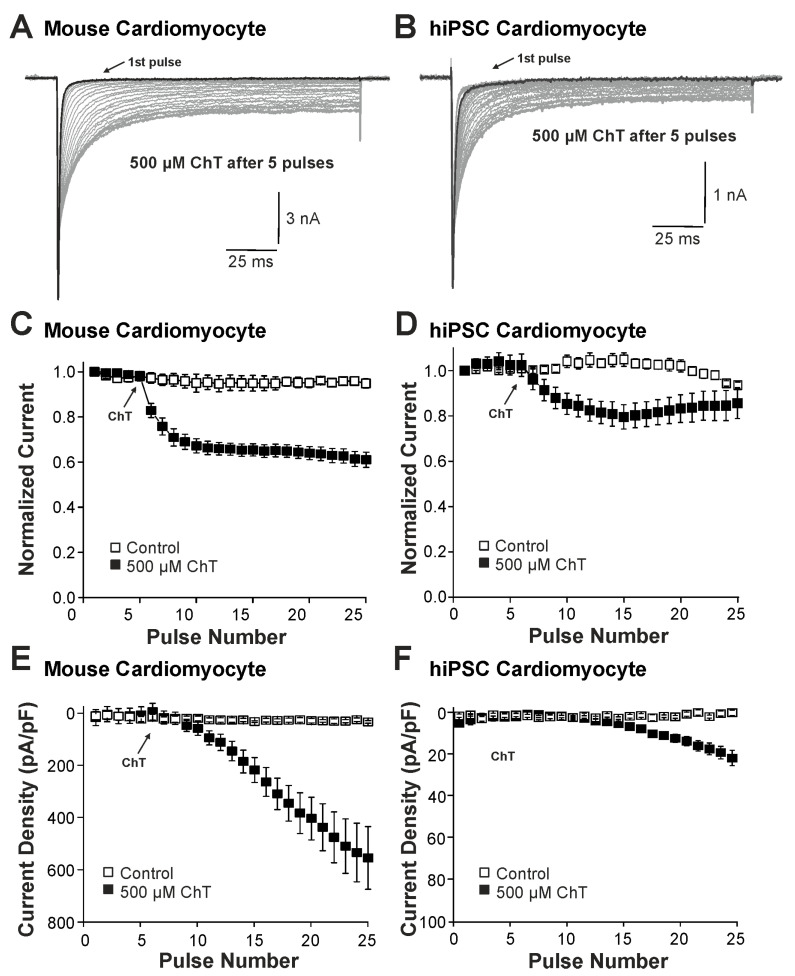
Distinct effects on Na^+^ currents in mouse cardiomyocytes and hiPSC-CMs: (**A**,**B**). Activation of Na^+^ currents in cardiomyocytes (**A**) or hiPSC-CMs (**B**) at 0.1 Hz and a holding potential of −120 mV. While peak current amplitudes declined, non-inactivating Na^+^ currents emerged upon application of 500 µM ChT. (**C**,**D**). Development of Na^+^ current peak amplitudes following application of control solution or 500 µM ChT on mouse cardiomyocytes (**C**) hiPSC-CMs (**D**). Current amplitudes were normalized to the amplitude of the first current evoked prior application of ChT. (**E**,**F**). Current densities of late Na^+^ currents following application of control solution or 500 µM ChT on cardiomyocytes (**E**) or hiPSC-CMs (**F**). The amplitudes of late currents were determined between 40 and 60 ms.

**Figure 2:**
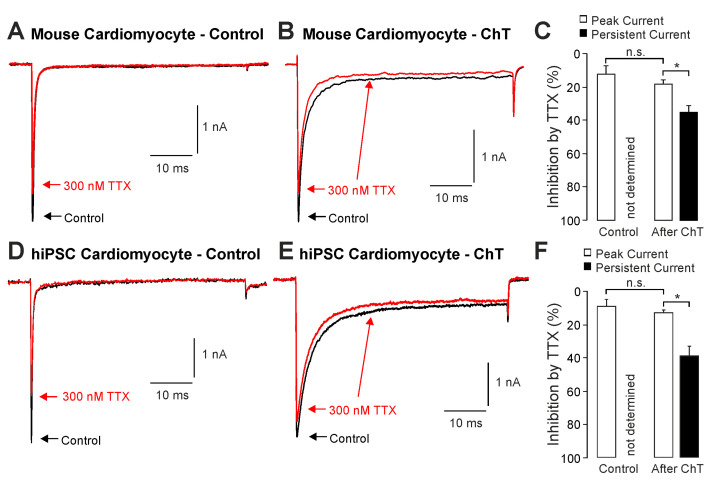
Inhibition of ChT-induced late Na^+^ currents by TTX: (**A**,**B**). Representative current traces displaying activation of Na^+^ currents in mouse cardiomyocytes before (**A**) and after application of 500 µM ChT (**B**). Application of 300 nM TTX (red traces) resulted in partially reduced amplitudes of peak currents as well as late Na^+^ currents. (**C**). Bar graphs displaying inhibition of peak currents as well as late Na^+^ currents by TTX before and after application of ChT. (**D**,**E**). Representative current traces displaying activation of Na^+^ currents in hiPSC-CMs before (**A**) and after application of 500 µM ChT (**B**). Application of 300 nM TTX (red traces) resulted in partially reduced amplitudes of peak currents as well as late Na^+^ currents. (**F**). Bar graphs displaying inhibition of peak currents as well as late Na^+^ currents by TTX before and after application of ChT. Data are presented as mean ± S.E.M. * = *p* < 0.05. n.s. = not significant.

We first examined the effect of ChT (500 µM) on Na^+^ currents in ventricular mouse cardiomyocytes and in hiPSC-CMs. As is demonstrated in Figure 1A–D, ChT induced a reduction in peak current amplitudes accompanied with late non-activating currents in both mouse cardiomyocytes and in hiPSC-CMs. Interestingly, Na^+^ currents in hiPSC-CMs partly recovered from ChT-induced inhibition at the end of the experimental protocol (Figure 1D). It was also evident that while mouse cardiomyocytes produced large ChT-induced late currents measuring between 40 and 60 ms (Figure 1E, n = 17), hiPSC-derived-CMs produced smaller late currents (Figure 1F, n = 10, *p* < 0.001, unpaired *t*-test). In order to investigate if TTX-sensitive brain-type Na^+^ channels are relevant for these ChT-induced late currents, 300 nM TTX was applied following application of either a control solution or ChT. This concentration of TTX fully inhibits brain-type α-subunits, but not Nav1.5 [1,25]. In mouse cardiomyocytes, TTX partly inhibited peak Na^+^ currents in both untreated (Figure 2A, 12 ± 5%, n = 7) and ChT-treated (Figure 2B, 18 ± 8%, n = 7) cells. This inhibition was not significantly different between both groups (Figure 2C, one-way ANOVA, *p* = 0.533, F = 9.15118). However, the ChT-induced late currents exhibited a stronger TTX-induced inhibition as compared to peak currents (Figure 2C, 37 ± 11%, one-way ANOVA, *p* = 0.018, F = 9.15118). An almost identical pattern was observed in hiPSC-CMs; e.g., TTX inhibited ChT-induced late Na^+^ currents significantly stronger than peak current amplitudes (Figure 2D–F, one-way ANOVA, *p* = 0.014, F = 4.70819, n = 9 each group). These data indeed suggest that brain-type Na^+^ channels may be relevant for ChT-induced late Na^+^ currents in cardiomyocytes.

**Figure 3:**
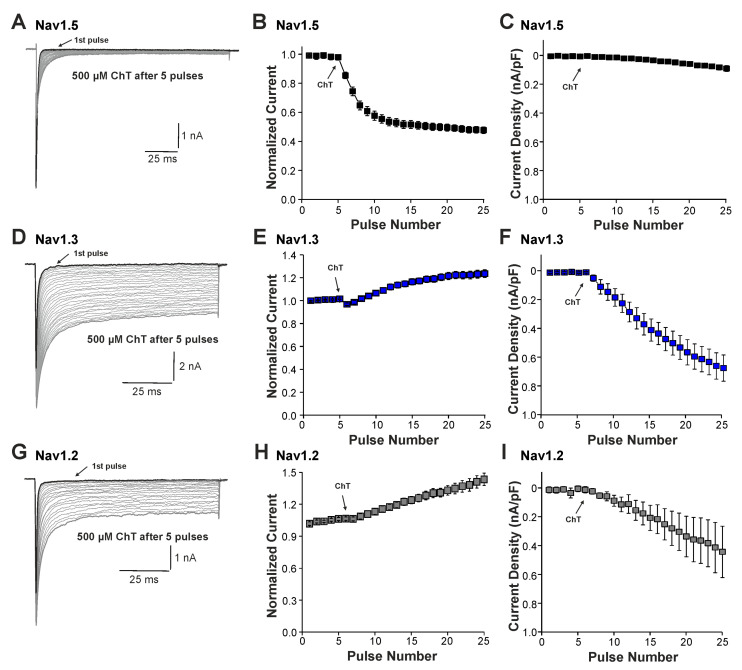
ChT-induced effects on recombinant α-subunits: (**A**,**D**,**G**). Traces displaying activation of Nav1.5 (**A**), Nav1.3 (**D**), or Nav1.2 (**G**) expressed in HEK 293 cells held at −120 mV. The first and last current traces are marked as bold black lines. (**B**,**E**,**H**). Development of Na^+^ current peak amplitudes following application of 500 µM ChT. Current amplitudes were normalized to the amplitude of the first current evoked prior application of ChT. (**C**,**F**,**I**). Development of late Na^+^ currents following application of 500 µM ChT. In all figures, data are presented as mean ± S.E.M.

**Figure 4:**
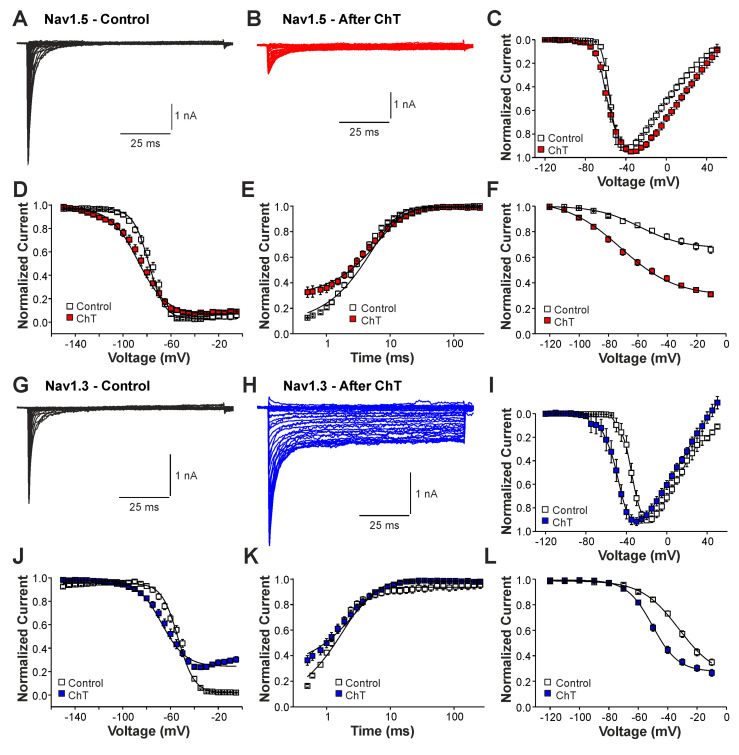
Differential effects of ChT on Nav1.5 and Nav1.3: (**A**,**B**). Current traces displaying voltage-dependent activation of Nav1.5 in control solution (**A**) or after application of 500 µM ChT (**B**). (**C**). Plot displaying normalized current–voltage dependencies of Nav1.5 in control solution and after application of ChT. Currents were normalized to the peak current. (**D**). Steady-state fast inactivation of Nav1.5 in control solution or after treatment with ChT. Normalized amplitudes were plotted against the pre-pulse potential and data were fitted with the Boltzmann equation. (**E**). Recovery from fast inactivation of Nav1.5 in control solution and after treatment with ChT. Normalized amplitudes were plotted against the corresponding interval duration. The lines are best fits of the normalized data calculated with an exponential potential. (**F**). Slow inactivation of Nav1.5 in control solution or after treatment with ChT. Normalized amplitudes were plotted against the membrane potential. Because Nav1.5 does not undergo substantial slow inactivation in control (<50%), the data could not be fitted with a Boltzmann equation. Therefore, a B-spline was drawn between the data points in order to guide the eye. (**G**,**H**). Current traces displaying voltage-dependent activation of Nav1.3 in control solution (**G**) or after application of 500 µM ChT (**H**). (**I**). Plot displaying normalized current–voltage dependencies of Nav1.3 in control solution and after application of ChT. (**J**). Steady-state fast inactivation of Nav1.3 in control solution or after treatment with ChT. Normalized amplitudes were plotted against the membrane potential and data were fitted with the Boltzmann equation. (**K**). Recovery from fast inactivation of Nav1.3 in control solution and after treatment with ChT. The lines are best fits of the normalized data calculated with an exponential potential. (**L**). Slow inactivation of Nav1.3 in control solution or after treatment with ChT. Normalized amplitudes were plotted against the membrane potential and data were fitted with the Boltzmann equation. All data are presented as mean ± S.E.M.

We next examined the effects of ChT on recombinant Nav1.2, Nav1.3, and Nav1.5 channels expressed in HEK 293 cells. As is demonstrated in Figure 3A–C, 500 µM ChT induced a strong reduction in peak current amplitudes accompanied with only minute late non-activating currents in cells expressing Nav1.5 (n = 38). The reduction in peak amplitudes was significantly stronger on Nav1.5 as compared to Na^+^ currents mouse in (*p* < 0.001, unpaired *t*-test). In contrast, 500 µM ChT induced an increase in peak Na^+^ current amplitudes in the cells expressing Nav1.3 (Figure 3C,D, n = 36). Even more prominent was the difference for ChT-induced late Na^+^ currents; e.g., Nav1.3 generated large late Na^+^ currents following treatment with ChT (Figure 3C,E). Na^+^ currents in cells expressing Nav1.2 displayed an almost identical pattern; e.g., ChT induced increased peak current amplitudes and large non-inactivating late currents (Figure 3F–H, n = 7). These data confirm that TTX-sensitive subunits like Nav1.3 and Nav1.2 are more prone to produce ChT-induced late Na^+^ currents as compared to Nav1.5. As these data also indicate that these α-subunits may exhibit fundamentally different ROS sensitivities, we next studied further ChT-induced effects on Nav1.5 and Nav1.3. While ChT again strongly inhibited peak current amplitudes of Nav1.5, the voltage-dependency of activation did not seem to be significantly shifted (Figure 4A–C, n = 10). We observed a rather prominent ChT-induced shift in fast inactivation (Figure 4D, control V1/2: −79 ± 0.7 mV, ChT V1/2: −88 ± 1.0 mV, n = 14), but recovery from fast inactivation was only marginally changed (Figure 4E, control τ: 5.3 ± 0.4 ms, ChT τ: −6.8 ± 0.3 ms, n = 12). Finally, ChT strongly increased the slow inactivation of Nav1.5 (Figure 4F, n = 13). We performed identical experiments on HEK 293 cells expressing Nav1.3. As is demonstrated in Figure 4G,H, 500 µM ChT potentiated currents produced by Nav1.3, and it induced a clear hyperpolarizing shift in the IV curve of Nav1.3 (Figure 4I, n = 13). Similar to Nav1.5, ChT also induced a slightly shifted fast inactivation (Figure 4J, control V1/2: −54 ± 0.6 mV, ChT V1/2: −67 ± 1.1 mV, n = 13), recovery from fast inactivation (Figure 4K, control τ: 1.9 ± 0.1 ms, ChT τ: −2.9 ± 0.1 ms, n = 9), and slow inactivation (Figure 4L, control V1/2: −32 ± 1.8 mV, ChT V1/2: −50 ± 0.5 mV, n = 7). These data suggest that, at a minimum, oxidation induced by ChT strongly promotes the activation of Na1.3, but reduces the availability of Nav1.5.

### 2.2. Late Na^+^ Currents Induced by UVA-Light

**Figure 5:**
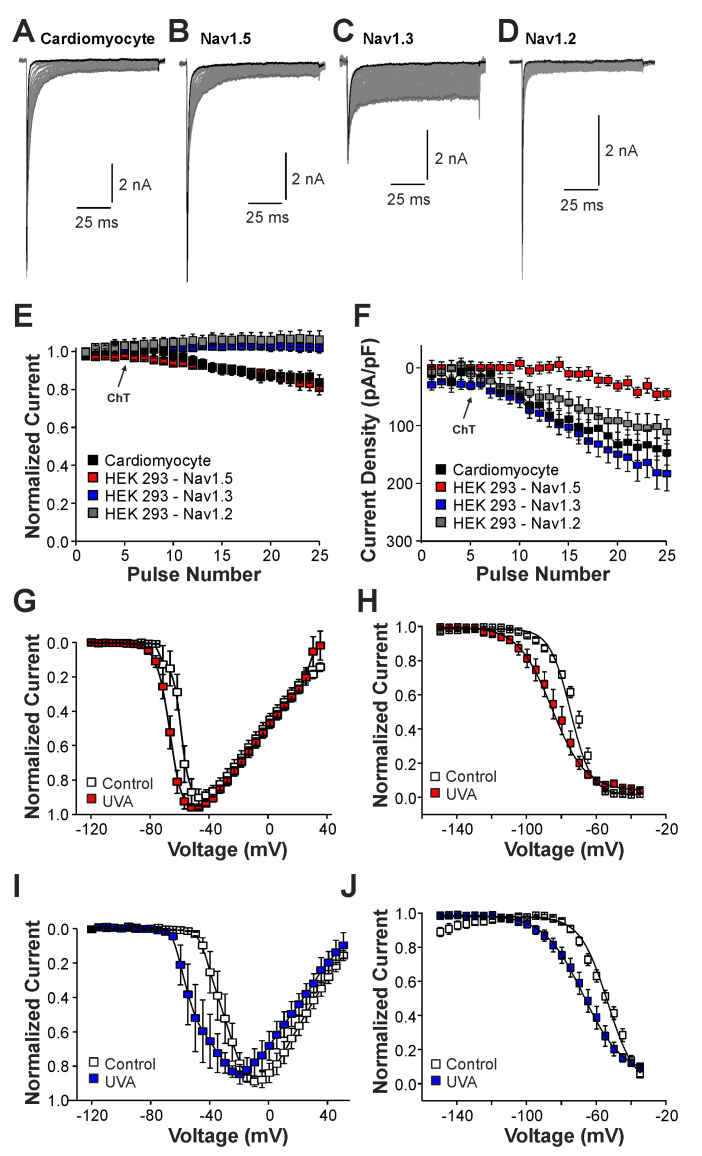
UVA-light induced effects on Nav1.5 and Nav1.3: (**A**–**D**). Current traces displaying activation of Na^+^ currents in cardiomyocytes (**A**) or HEK 293 cells with Nav1.5 (**B**), Nav1.3 (**C**), or Nav1.2 (**D**). Non-inactivating Na^+^ currents emerged upon application of UVA-light. The first and last current traces are marked as bold black lines. (**E**). Development of Na^+^ current peak amplitudes following application of UVA-light. (**F**). Development of late Na^+^ currents following application of UVA-light. In (**D**,**E**), current amplitudes were normalized to the amplitude of the first current evoked prior application of UVA-light. (**G**,**I**). Current–voltage dependencies of Nav1.5 (**G**) or Nav1.3 (**I**) in control solution and after application of UVA-light. (**H**,**J**). Steady-state fast inactivation of Nav1.5 (**H**) and Nav1.3 (**J**) in control solution or after treatment with UVA-light. Normalized amplitudes were plotted against the membrane potential and data were fitted with the Boltzmann equation. All data are presented as mean ± S.E.M.

We next studied the induction of late Na^+^ currents by irradiation of the cells with UVA-light (380 nm). UVA-light induces an intracellular accumulation of ROS, and it was previously demonstrated to modify Nav1.5 and other Na^+^ channels [22]. In mouse cardiomyocytes, UVA-light induced a small reduction in peak current amplitudes accompanied by a modest increase in late Na^+^ currents (Figure 5A,E,F, n = 7). Similar effects were observed in HEK 293 cells expressing Nav1.5 (Figure 5B,E,F, n = 15), i.e., a decrease in current peak amplitudes and the devolvement of small late currents. In contrast, cells expressing Nav1.3 (Figure 5C, n = 19) or Nav1.2 (Figure 5D, n = 8) exhibited increasing peak current amplitudes and late currents during exposure to UVA-light (Figure 5E,F). Similar to ChT, UVA-light also induced a shift in the voltage-dependency of activation on both Nav1.5 and Nav1.3 (Figure 5G,I, n = 8 and 9, respectively), but again this effect seemed to be more pronounced on Nav1.3. Furthermore, both Nav1.5 and Nav1.3 exhibited a strongly shifted fast inactivation following irradiation by UVA-light; Nav1.5 (Figure 5H, control V1/2: −75 ± 0.7 mV, UVA V1/2: −86 ± 0.6 mV, n = 9), Nav1.3 (Figure 5J, control V1/2: −54 ± 1.7 mV, ChT V1/2: −68 ± 0.7 mV, n = 6). Thus, even if UVA-light-induced effects were in general less pronounced than ChT-induced effects, these data confirm that Nav1.5 is less prone to generate oxidation-induced late currents as compared to Nav1.3.

### 2.3. Modulation of Nav1.5 and Nav1.3 by a Cysteine-Selective Oxidant

**Figure 6:**
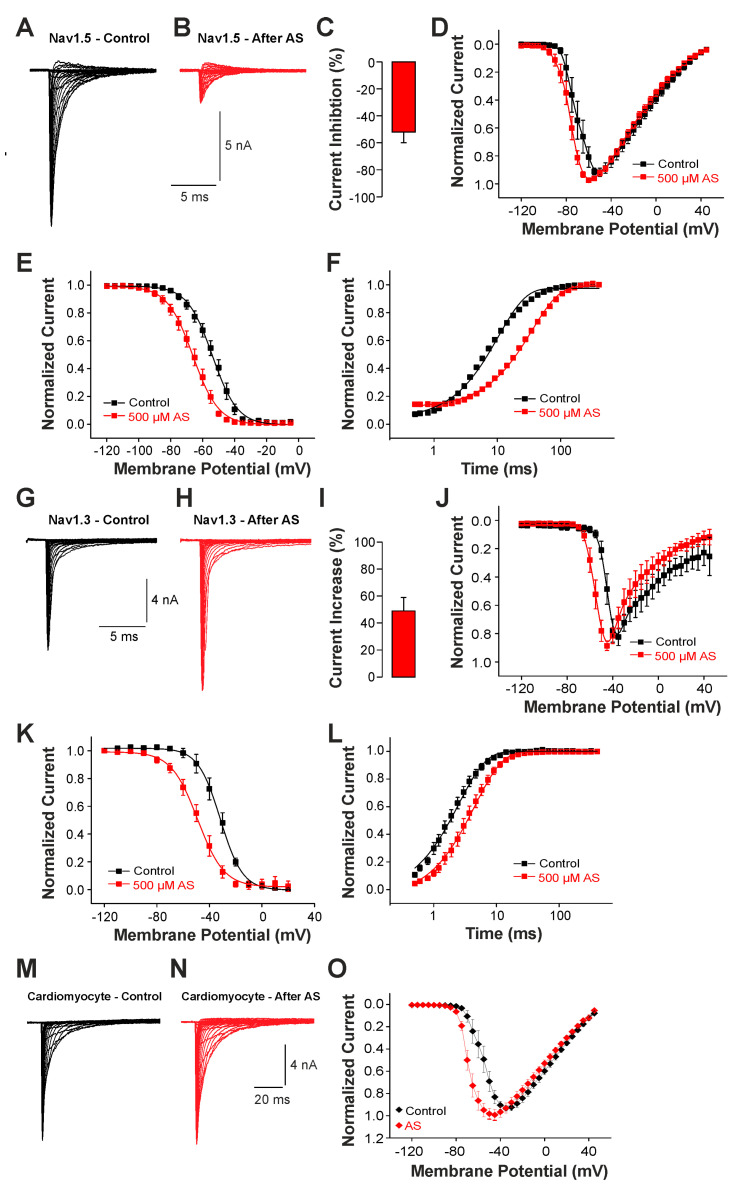
Different effects of AS on Nav1.5 and Nav1.3: (**A**,**B**,**G**,**H**). Voltage-dependent activation of Nav1.5 (**A**,**B**) or Nav1.3 (**G**,**H**) in control solution or after application of 500 µM AS. (**C**,**I**) Bar graphs displaying the average inhibition of Nav1.5 (**C**) and potentiation of Nav1.3 induced by 500 µM AS (**I**) in control solution and after application of AS. (**D**,**J**) Normalized current-voltage dependencies of Nav1.5 (**D**) and Nav1.3 (**J**) in control solution and after application of AS. (**E**,**K**) Steady-state fast inactivation of Nav1.5 (**E**) and Nav1.3 (**K**) in control solution or after treatment with 500 µM AS. Normalized amplitudes were plotted against the membrane potential and data were fitted with the Boltzmann equation. (**F**,**L**) Recovery from fast inactivation of Nav1.5 (**F**) and Nav1.3 (**L**) in control solution and after treatment with 500 µM AS. Normalized amplitudes were plotted against the corresponding interval duration. The lines are best fits of the normalized data calculated with an exponential potential. All data are presented as mean ± S.E.M. (**M**,**N**) Current traces displaying voltage-dependent activation Na^+^ currents in cardiomyocytes before (**M**) and after application of 500 µM AS (**N**). (**O**) Current-voltage dependencies of Na^+^ currents in cardiomyocytes examined in control solution and after treatment with AS.

While these findings suggest a role for brain-type Na^+^ channels like Nav1.3 for the generation of late Na^+^ currents following rather extreme pathophysiological conditions associated with strong oxidative stress, we finally examined the effect of nitroxyl (HNO) on Na^+^ currents. HNO induces positive cardio-vascular properties and is regarded as a potential agent for the treatment of acute heart failure [28]. The underlying mechanisms of HNO-induced effects involve an oxidation of cysteine residues resulting in covalent bonding and/or formation of reversible disulfide bonds [29]. Thus, unlike the unselective oxidants ChT and UVA-light known to induce late Na^+^ currents, we now examined the effects of a cysteine-selective oxidant on channel gating. As HNO itself is extremely unstable, we analyzed the effect of 500 µM Angelis Salt (AS), a donor of HNO. The pronounced instability of HNO only allows the active substance to modify the Na^+^ channel for a few seconds. Therefore, we did not examine the possible slow development of late currents. Instead, we investigated the effects of HNO on peak current amplitudes and the voltage dependencies of activation and fast inactivation. Currents were induced by depolarizing pulses ranging from −120 to +45 mV in increments of 10 mV. As is demonstrated in Figure 6A,B, cells expressing Nav1.5 generated Na^+^ currents that were strongly reduced following treatment with AS. The average peak current amplitude was reduced by 52 ± 8% (Figure 6C, n = 10, *p* < 0.001 paired *t*-test). When plotting the normalized peak current amplitudes against the corresponding voltage of the test-pulses, we also observed a small leftward shift in the voltage dependency of activation (Figure 6D). We next explored if AS also modifies the steady-state fast inactivation of Nav1.5. As is demonstrated in Figure 6E, AS induced a rather strong shift in the midpoint of steady-state fast inactivation from V1/2 −54 ± 0.3 mV to −66 ± 0.4 mV (n = 7). Finally, we examined the effects of AS on recovery from fast inactivation. Application of 500 µM AS resulted in a strongly decelerated recovery from inactivation, e.g., the time constant increased from 10 ± 0.3 ms to 35 ± 1.2 ms (n = 8, Figure 6F). We proceeded with identical experiments on Nav1.3. As is demonstrated in Figure 6G,I, AS induced an increase in current amplitudes by 49 ± 10% (n = 12, *p* < 0.01 paired *t*-test). The voltage dependency of activation of Nav1.3 displayed a marked shift toward more hyperpolarized potentials following treatment with HNO (Figure 6J). AS also induced a strong shift in the midpoint of fast inactivation of Nav1.3, from −32 ± 0.4 mV to −49 ± 0.5 mV (Figure 6K, n = 10). Furthermore, recovery from the fast inactivation of Nav1.3 was slowed down by AS (from 2.5 ± 0.08 ms to 4.5 ± 0.1 ms, n = 10, Figure 6L). These data thus suggest that also the cysteine-selective oxidant HNO exerts differential effects on Nav1.5 (e.g., inhibition) and Nav1.3 (e.g., potentiation). We finally examined the effect of 500 µM AS on voltage-dependent activation of Na^+^ currents in cardiomyocytes. As is demonstrated in Figure 6M,N, AS did not induce any obvious effects on the peak amplitudes of Na^+^ currents in cardiomyocytes (n = 10, *p* = 0.298, paired *t*-test). However, the current–voltage plot revealed a clear leftward shift in the voltage dependency of activation (Figure 6O, n = 10). There is little doubt that Nav1.5 is the predominant Na^+^ channel in these mouse cardiomyocytes; e.g., we were surprised that HNO did not inhibit Na^+^ currents. The most likely reason for the lack of a HNO-induced current inhibition may be that the effective concentration of HNO was too low. Saying this, our experiments with higher concentrations of AS were not successful.

## 3. Discussion

The physiological role of brain-type α-subunits in the heart is yet to be exactly defined, but we also have a limited understanding of their pathophysiological roles as well as their utility as putative targets for novel cardiac pharmaceuticals. Previous reports suggested that brain-type subunits may account for certain arrhythmias [9,10,11]. Furthermore, they were also reported to be relevant for the generation of late Na^+^ currents in cardiomyocytes from untreated dogs and rats as well as from dogs with a model for heart failure [12,13,14,15,30]. While Mishra and colleagues suggested that Nav1.1 generates late Na^+^ currents in cardiomyocytes following a chronic model on dogs for failing hearts [12], Biet et al. found that TTX-sensitive isoforms generate ~50% of late Na^+^ currents in “healthy” dog cardiomyocytes [15]. Here, we demonstrate for the first time that brain-type α-subunits seem to account for a relevant part of late Na^+^ currents acutely occurring following strong oxidative stress. We show that neonatal mouse cardiomyocytes as well as hiPSC-CMs produce large non-inactivating Na^+^ currents oxidation, and that TTX inhibits 30–40% of these late currents. Accordingly, there was a striking difference between Nav1.5 as compared to Nav1.3 and Nav1.2 regarding their redox-sensitivities and thus their potential to generate ROS-induced late Na^+^ currents. While Nav1.5 exhibits a strong peak current reduction and only small non-activating currents following oxidation, Nav1.3 and Nav1.2 were even potentiated and generated large non-inactivating currents. To this end, it is important to note that we do not know if Nav1.1 and Nav1.6, e.g., two other brain-type isoforms expressed in heart, display the same properties as Nav1.3 and Nav1.2 in this regard. The fact that Nav1.1, Nav1.3, and Nav1.6 display very high sequence homologs makes it seem unlikely that they exhibit a Nav1.5-like phenotype regarding redox sensitivity [1], and the pore cysteine residue being responsible for oxidant-induced inhibition of Nav1.5 is not conserved in any other isoforms. Other than this pore cysteine residue in Nav1.5, we currently also do not know how different ROS-sensitivities among Nav-subunits are encoded. It seems likely that not yet identified cysteine or methionine residues in these complex proteins account for ROS-induced effects as well, but it is possible that differences in gating properties, membrane trafficking, or even associating molecules are also relevant.

Previous studies found that ChT also induces prominent late currents and an increase in channel availability on the subunits Nav1.4, Nav1.7, and Nav1.8 [18,26]. It was also demonstrated that recombinant Nav1.5 channels exposed to oxidants or UVA-light exhibit reduced peak currents, but a hampered fast activation resulting in non-inactivating late currents [22]. While the same study also described similar effects on Nav1.5 induced by ChT, they did not report on significant differences between Nav1.5 and Nav1.4 nor on Na^+^ currents in the neuronal cell line GH3 [22]. UVA-light was also reported to induce non-inactivating Na^+^ currents in guinea pig ventricular cardiomyocytes [31]. The authors of that study applied 100 µM TTX in order to validate that these late currents were indeed generated by Na^+^ channels. This high concentration of TTX inhibits all α-subunits and thus does not discriminate between TTX-sensitive and TTX-resistant ones. Oxidant-induced late Na^+^ currents in cardiomyocytes have also been demonstrated in several early studies using ChT, H_2_O_2_, or even hypoxia, but none of the studies explored the possible contribution of TTX-sensitive brain-type subunits [32,33,34]. Considering the general view that inhibition of late Na^+^ currents can reduce myocardial ischemia/reperfusion injury [35], our data suggest that substances preferentially inhibiting brain-type subunits may have cardio-protective properties. Indeed, TTX was demonstrated to reduce ischemic/reperfusion injury in Langendorff-perfused rat hearts [36]. However, only high concentrations of TTX were effective in that study. Thus, it is possible that Nav1.5 was also inhibited. Kirchhof and colleagues showed that selective activation of the brain-type Na^+^ channel Nav1.3 can increase cardiac contractility without provoking arrhythmia [7]. In this context, our data suggest that Nav1.3 is a feasible target of the positive inotropic agent HNO. Thus, HNO may exert its positive inotropic effect—in part—by increasing the sodium influx via Nav1.3. A higher subsarcolemmal sodium concentration culminates in an elevation of sarcoplasmic reticulum Ca^2+^ release and thereby to an increased cardiac contractility [37]. Furthermore, there is evidence that the subsarcolemmal sodium concentration could be a direct regulator of cardiac contractility, a possible mechanism for the increase in cardiac contractility through the selective activation of TTX-sensitive brain-type Na^+^ channels [38]. In contrast to ChT and most endogenous ROS, nitroxyl selectively targets cysteine residues leading to covalent binding or the formation of a reversible disulfide [27]. Thus, agents that selectively modify thiols may alter the properties of different voltage-gated Na^+^ channels without provoking persistent non-inactivating currents. However, a reduction in Na^+^ currents due to oxidative stress may lead to a conduction block that potentiates re-entry-type ventricular arrhythmias [21,39]. The hydrogen sulfide donor NaHS was shown to increase Na^+^ currents generated by Nav1.5 expressed in human jejunum smooth muscle cells via a redox-dependent mechanism [40]. In contrast, the hydrophilic substance thimerosal inhibits both Nav1.4 and Nav1.5, but also alters steady-state inactivation and activation due to the formation of disulfide bridges and a reduced channel permeability [41,42]. The cysteine-selective oxidant methanethiosulfonate ethylammonium (MTSEA) was demonstrated to block Nav1.5 by modifying the cysteine residue C373 located in the outer channel pore [23]. When C373 was replaced with a tyrosine, the MTSEA-induced block was diminished. The lack of pro-arrhythmic properties of HNO suggests that it probably does not substantially reduce the total cardiac Na^+^ currents when applied systemically.

This study has several substantial limitations. First, we compared functional properties between recombinant channels with Na^+^ currents of cardiomyocytes from neonatal mice as well as of hiPSC-SMs. It is important to note that this approach did not allow us to judge if certain findings may be due to species-specific differences, or even the expression splice variants of Nav1.5 in embryonic cardiomyocytes, or if the neonatal cardiomyocytes display a fundamentally different ROS sensitivity as compared to adult cells or HEK 293 cells. Therefore, our data are not conclusive in many ways, but they encourage further studies on more intact preparations.

To conclude, our in vitro data suggest that the predominant cardiac subunit Nav1.5 is inhibited by oxidants and therefore generates relatively small ROS-induced non-inactivating currents. Instead, brain-type subunits including Nav1.3 produce large oxidant-induced late currents that seem to be relevant for the oxidant-induced late currents generated in cardiomyocytes. If this hypothesis holds true, Nav1.3 and other brain-type subunits may be suitable targets for the treatment of arrhythmias occurring due to hypoxia or ischemia. On the other hand, our data also suggest that the positive inotropic effect induced by nitroxyl may involve the sensitization of Nav1.3 and other brain-type subunits.

## 4. Materials and Methods

### 4.1. Cell Culture and Transfection Procedure

Cells were cultured under standard tissue culture conditions (5% CO_2_, 37 °C) in cell culture flasks and split three times a week. HEK 293 cells stably expressing human Nav1.5 or rat Nav1.3 were cultured and used as described previously [31]. Briefly, cells were cultured in Dulbecco’s modified Eagle medium (DMEM, GIBCO-Invitrogen, Darmstadt, Germany) supplemented with 10% heat-inactivated fetal bovine serum (FBS, Biochrom, Berlin, Germany), 1% penicillin/ streptomycin (GIBCO-Invitrogen, Darmstadt, Germany), and 1% G418 (GIBCO-Invitrogen, Darmstadt, Germany). Site-directed mutagenesis was performed to generate hNav1.5-C373S according to the instructions of the manufacturer with the QuikChange Lightning site-directed mutagenesis kit (Agilent, Waldbronn, Germany). p.C373S cDNA (2 µg) was transiently transfected into the HEK293 cells using jetPEI transfection kit (Polyplus-transfection^®^ SA, Illkirch, France).

### 4.2. Mouse Cardiomyocytes

Ventricular cardiomyocytes were isolated by enzymatic digestion of E16.5 fetal murine hearts. Female mice were sacrificed by cervical dislocation and embryos were harvested in PBS, decapitated, hearts dissected, and all tissue but the ventricles removed. The ventricular tissue was washed to remove all blood, cut into pieces, and digested in 0.5% collagenase IV/0.0125% trypsin. Cardiac fibroblasts were removed by pre-plating gelatin-coated dishes. Cardiomyocytes were maintained on gelatin-coated glass slides at 37 °C and 5% CO_2_ for 2 days before measurement and cultivated in DMEM, 20% M199 (GIBCO-Invitrogen, Darmstadt, Germany), 10% horse-serum (GIBCO-Invitrogen, Darmstadt, Germany), FBS 10%, and 1% penicillin/streptomycin. Hannover Medical School is a holder of PHS-approved animal welfare assurance (A5919-01) in compliance with the guide for the Care and Use of Laboratory Animals published by the US National Institutes of Health (NIH Publication, 8th Edition, 2011). All animal work conducted for this study was approved by A. Bleich, state head of the animal facility at Medizinische Hochschule Hannover, and performed according to German legislation.

### 4.3. Chemicals and Solutions

Angeli’s salt (Biomol, Hamburg, Germany) was diluted in the external solution to the desired concentrations directly before recordings. According to the information of the distributor of AS, 500 µM AS should theoretically produce ~270 µM HNO. Chloramine T was obtained from Sigma-Adrich, and Tetrodotoxin was obtained from Alomone Labs (Jerusalem, Israel). The extracellular solution contained (mmol/L) 140 NaCl, 3 KCl, 1 CaCl_2_, 1 MgCl_2_, 10 HEPES, and 10 glucose. Adjustment of pH value to 7.4 was performed with TMA-OH (tetramethylamoniumhydroxide, Sigma-Aldrich, Taufkirchen, Germany). The internal solution contained (mmol/L) 140 CsF, 10 NaCl, 10 HEPES, and 1 EGTA. The pH value was adjusted to 7.4 with CsOH.

### 4.4. Electrophysiology and Data Acquisition

Whole-cell patch clamp experiments were conducted at room temperature (~20 °C) with an EPC10 amplifier and the Patchmaster v20x60 software (HEKA Instruments Inc., Bellmore, NY, USA). Data were analyzed with the Fitmaster software version 2x92 (HEKA Instruments Inc.) and curve fitting was performed with Origin 6.0 (Microcal Software, Northampton, MA, USA). Patch pipettes were fabricated with borosilicate glass capillaries (Science Products, Hoffenheim, Germany). The resulting resistance was 1.5–2.5 MΩ. Currents were filtered at 5 kHz and sampled at 20 kHz. The series resistance was compensated by 60–80%. Linear leak subtraction, based on resistance estimates from four hyperpolarizing pulses was applied after the depolarization test potential. Cells were used for further evaluation only if they had an initial seal > 1 Gigaohm, a stable leak current < −200 pA throughout the recording, and an access resistance < 10 Megaohm. Experiments were started > 3 min after the whole-cell mode was established. Normalized steady-state inactivation curves were fitted with the Boltzmann function: I/Imax = 1/(1 + exp ((V50-inac + V)/Kinac)) (Imax = peak sodium current, V50-inac = the potential at which 50% of channels are inactivated, V = potential of the pre-pulse, and kinac = slope factor). For all experiments, the holding potential was −120 mV. The protocols performed in the study were designed as follows:

1. Development of amplitudes of peak currents and late currents: Currents were evoked by 25 × 100 ms long pulses to 0 mV applied every 10 s. The amplitudes of the late currents were measured between 40 and 60 ms. Due to the development of late currents, the time constant for current inactivation increased. This time (τ) constant was calculated by single exponential (y = y_0_ + A_1_*e*^−x/τ^) fits of the inactivation phase of each cell investigated.
2. Voltage-dependent activation: Currents were evoked by 100 ms long test-pulses ranging from −120 to 45 mV in steps of 5 mV.
3. Fast inactivation was induced by 50 ms long inactivating pulses ranging from −120 to −50 mV applied before the test-pulse to 0 mV.
4. Slow inactivation was induced by 10 s long inactivating pulses ranging from −120 to −10 mV. A 100 ms long pulse to −120 mV allowing recovery from fast inactivation was applied before the test-pulse to 0 mV.
5. Recovery from fast inactivation: Nav1.5 was inactivated by a 50 ms long pre-pulse to 0 mV. The fraction of available channels was examined with a consecutive test-pulse to 0 mV applied after variable intervals at −120 mV.

### 4.5. Data and Statistical Analysis

All data are expressed as mean ± standard error of mean (S.E.M.). Statistical analyses were performed with Origin 6.0 with appropriate tests as indicated in the text.

*4.6. hiPSC-CMs Cultivation, Directed Cardiac Differentiation, and Maturation*

Directed cardiac differentiation of hiPSC cell line: Phoenix [43] was performed in suspension culture as described [44,45]. For patch clamp experiment, hiPSC-CMs aggregates after ~14 to 18 days of differentiation were dissociated with the STEMdiff Cardiomyocyte Dissociation Kit (StemCell Technologies, Cologne, Germany) for 3 min. Cells were plated on fibronectin + 0.1% gelatine-coated, round glass coverslips in medium containing 80% IMDM + Glutamax (GIBCO-Invitrogen, Darmstadt, Germany), 20% fetal calf serum, 1 mM L-glutamine, 0.1 mM ß-mercaptoethanol, and 1% nonessential amino acids (all GIBCO-Invitrogen). The medium was supplemented with 10 µM Y-27632 (ROCK inhibitor) for the first 24 h. Thereafter, the medium was replaced for RPMI-1640+B-27 supplement (ThermoFisher Scientific, Waltham, MA, USA) and replenished every 2–3 days before patch clamping at ~21 to 25 days post cell seeding.

## Data Availability

All data created in this study are included in the manuscript.
